# The Technology Acceptance of a TV Platform for the Elderly Living Alone or in Public Nursing Homes

**DOI:** 10.3390/ijerph14060617

**Published:** 2017-06-08

**Authors:** Pedro C. Santana-Mancilla, Luis E. Anido-Rifón

**Affiliations:** 1 School of Telecommunications Engineering, University of Vigo, 36310 Vigo, Spain; lanido@gist.uvigo.es; 2 School of Telematics, University of Colima, Colima 28040, Mexico

**Keywords:** interactive television, seniors, elderly, homecare, technology adoption, public nursing homes

## Abstract

In Mexico, many seniors are alone for most of the day or live in public nursing homes. Simple interaction with computer systems is required for older people. This is why we propose the exploration of a medium well known by seniors, such as the television (TV). The primary objective of this study is to improve the quality of life of seniors through an easier reminder system, using the television set. A technological platform was designed based on interactive television, through which seniors and their caregivers can have a better way to track their daily activities. Finally, an evaluation of the technology adoption was performed with 50 seniors living in two public nursing homes. The evaluation found that the elderly perceived the system as useful, easy to use, and they had a positive attitude and good intention to use it. This helped to generate initial evidence that the system supported them in achieving a better quality of life, by reminding them to take their medications and increasing their rate of attendance to their medical appointments.

## 1. Introduction

### 1.1. Background

Technological advancementsin information and communication technologies (ICT) play a fundamental role in all knowledge and service areas, but especially in health care, with the emergence of highly complex devices for home healthcare which helps service providers to communicate in-home with their patients [1].

Home care is one of the most accepted services in countries like United States, Spain, England, and Germany. It is estimated that in the U.S., 20,000 professionals provide health care at home to 8 million individuals. These numbers will surely rise as life expectancy increases. Additionally, the growing number of older people has resulted in an increase in chronic diseases (heart attacks, diabetes and renal disorders), cognitive impaired individuals [2], and sensory and motor function impairments, thus compromising elderly independence and quality of life.

Aging, or the biological degeneration of body functions, is related to the accumulation (during the lifetime elapsed) of genetic faults in cells and organs; in some cases, augmented by additional factors, such as: diet, lifestyle and diseases [3]. Additionally, ageing can be understood in terms of psychological changes associated with sensory, cognitive and psychomotor skills, such as: vision, hearing, and cognition [4,5,6,7,8,9,10,11].

As a result, while many people will experience small reductions in their skills, others will become fragile and impaired.

### 1.2. Seniors and Interactive TV

Simple interaction with computer systems is required for older people [12]. They are often unable to use technological tools because these tools were not designed to support the natural changes that older adults face due to ageing [13], and elderly people typically show rejection of new technologies at home, either because the lack of support to their natural disabilities, or the lack of perceived utility [14].

For the aforementioned, we propose to explore a medium well known by seniors, such as the television set (TV). TV is a standard device with an extensive home penetration, and has impacts in practically all scopes of information, entertainment and education delivery [15,16]. Thus, TV plays a primary role in society [17].

The combination of traditional television and interactive applications for use in a television set [18], and the development of TV contents that include interaction and digital improvements, are called Interactive Television (iTV). The role of TV viewers has evolved from a passive role to an active one [14], thus allowing the proposal of iTV as a means for providing health care to elderly people. Several studies [15,16] demonstrate that:

- Seniors are a broad group with a high level of TV program consumption but little or no access to the information society.
- Seniors are a group with more free time, which is mainly used to read news or watch TV.

As the TV is a natural device for these groups of people, the learning gap for TV applications is reduced, and in the future, it will be possible to provide other services together with homecare, such as:

- Preventive medicine.
- Dietary treatments (cholesterol, diabetes, cardiovascular problems).
- Formative programming content with interaction from the elders.
- Electronic payments (useful for the groups that are not used to the Internet).
- Entertainment.
- Serious gaming (to support cognition).

This paper describes the user acceptance evaluation and the main characteristics of iTVCare, a technological platform based on interactive television to provide seniors with support for challenging everyday activities such as medication intake and reminders of medical appointments, to improve the quality of life of older adults in non-clinical settings, such the home and public nursing homes. The objective of this work was to obtain evidence about the acceptability of iTVCare by older adults, and to determine if a television set is suitable and accepted by older people, in order to help improve their quality of life in an age-friendly environment.

## 2. Methods

### 2.1. User Centered Design

This research used the User Centered Design [19], which is detailed below.

1. Specify the usage context: Identify people that will use the product, what their goals are in using it, and under which circumstances they will use it.
2. Specify requirements: Identify all the requirements and the users’ objectives that are needed for success with the product.
3. Produce design solutions: This part must follow an iterative process, starting from a low fidelity prototype to a high-fidelity prototype.
4. Evaluation: This stage must be (ideally) executed through tests with real users; this is the most important part of the process.

### 2.2. Usage Context and Requirements

The context usage for the developed platform was Colima, a state located in Mexico. The platform requirements are listed below.

We identified 500 elderly people and caregivers (*n* = 500) willing to share their experiences with us in answering questions in an interview oriented towards understanding the access to technology of Mexican seniors living in the State of Colima (65+ years). People of different gender, age, and living in different cities were included; the total number of older people living in Colima State was 58,728, and the sample had an error margin of 4.36%, and a confidence level of 95%.

Some of the more relevant results were: 99% of seniors have a television set, which indicates a high percentage of penetration in Mexican homes, and 1% reported lacking electrical power service. TV was widely used as an important part of daily activities, 85.86% watched TV for more than an hour a day, and 37.17% spent more than three hours watching television. Almost 30% did not have access to a computer or a cell phone, so they did not know how to use these types of devices.

Furthermore, older people tend to have problems interacting with new technology because it is not designed for their especial needs [20], therefore, the system must provide easy-to-use tools for the elderly to obtain greater control over their medical appointments and to provide reminders about medication intakes. 

Johnson and Finn [21] emphasize that poorly designed user interfaces tend to affect elderly people more seriously than young people. They propose to lessen these effects by designing user interfaces with these issues in mind, and therefore improving the user experience for this group.

As usability specialists, [21] compared younger and older participants and they found that older ones tended to:

- Take longer to learn new applications or devices.
- Take longer to complete tasks.
- Use different search strategies.
- Perform worse on tasks relying on memory.
- Be more distractible.
- Have a harder time dealing with errors.
- Make more erratic or accidental movements with the pointer.
- Make more input errors.
- Have more trouble hitting on-screen targets.

By designing our system considering all of the requirements previously mentioned, we will have an efficient system for reminders in the daily life of the elderly, which can also be managed by their caregivers.

### 2.3. Design and Requirements

The special needs of older people make it necessary to comply with design guidelines created to support their specific needs.

Some of these guidelines [21,22,23] were used to design a low-fidelity prototype of iTVCare. Next we summarize the more relevant features:

1. Minimize the number of steps: As the Human-TV Interaction system is mainly operational through a remote control and can be exasperating, we minimized the number of steps from the home screen to reach a given screen.
2. Use consistency: iTVCare offers the same set of options in the same order to facilitate recognition.
3. Reduce the information presented: Memory impairment means that elderly people need interfaces with no irrelevant information.
4. Clear indication of the current screen: As mentioned, memory impairment makes older adults more susceptible to getting lost in the application.
5. Use meaningful icons and labels: Because older adults are more likely to have vision problems, graphic symbols are beneficial so that the users do not need to struggle with words.

Additionally, we used several recommendations that are quite well known and accepted:

- Use a very large font type;
- Use an easy to read font family;
- Use mixed case;
- Leave plenty of space;
- Present few calls to action;
- Design error messages to be clear;
- Make it easy to correct input errors;
- Avoid the use of scroll;
- Use high contrast between elements of the user interface.

At this stage of the project, a heuristic evaluation study was performed through expert review to the iTVCare prototype to make sure that the requirements identified after the contextual study were included in a user-friendly manner to older adults. Heuristics were an efficient method for finding problems with the system, and allowed significant improvements in interaction with the software [17].

After addressing and resolving the findings of the heuristic evaluation, we proposed the creation of an improved platform of interactive television based on Google TV’s technology (now Android TV). It is an open platform, and enhances the experience of television viewers with the power of the Internet and proper applications developed for the platform [24]. Google TV is built on the Android operating system, also owned by Google. As a development platform, it includes Google Chrome and applications developed with Android Software Development Kit (SDK) that provides access to multimedia services, search capacity and the Application Programming Interface (API) of Google TV.

To provide a scalable platform, we used a component-based development process that uses software components as fundamental elements for developing applications [25]. Components are packages of executable software that expose their functionality through a well-defined and published interface [26]. The developed components are loosely coupled [27] to allow easily add new functionality to the system (e.g., E-government services) and authorize access to users that owners or users would like to be in contact with, like their caregivers.

**Figure 1:**
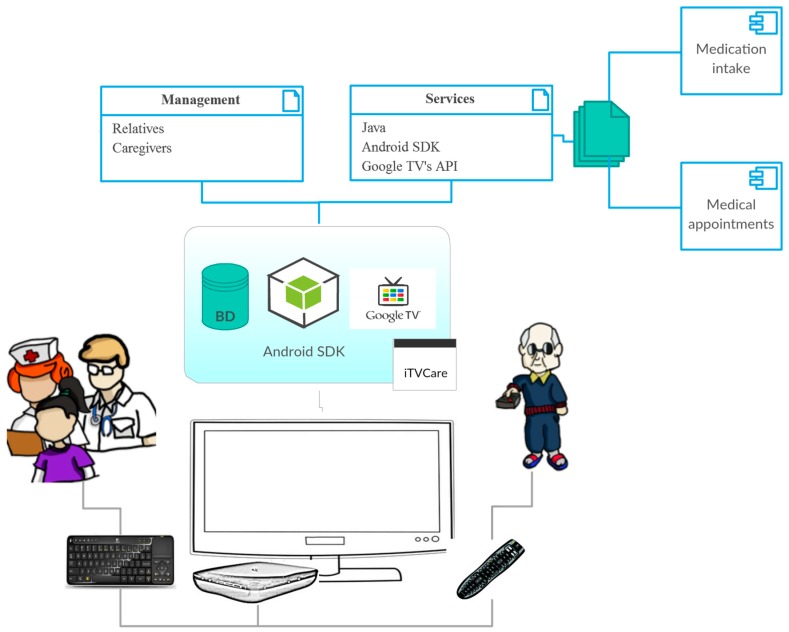
System architecture.

The main components of the system architecture (Figure 1) are described below:

- iTVCare: This layer represents the home system of the user, where the Google TV is installed.
- Database: This component stores information for medical appointments and medicine intakes.
- Television with Google TV: The television requires a device in charge of the reception and decoding of the TV signal, and the execution of the interactive applications that will be displayed on the television. This device is known as a set-top-box (STB).
- Android SDK: Contains the development tools that enable developers to create these applications, and the STB to execute the applications.
- Services: The services layer includes the components to be consumed by iTVCare.
- Medication intake: This component manages users’ medicine intakes, and generates alerts when it is time for a new intake.
- Medical appointments: This component manages appointments with doctors.
- Management: Finally, users will receive the information visually from the television and interact with the applications using the remote control and a keyboard. The users can be both older adults and their caregivers.

We envisioned that, to improve ageing-well process, our system needed to address the following aspects. Older people and their caregivers can contribute to add the information to the system, thus we considered interfaces suitable for usage by both the caregivers and the elders. We considered that, for older people, we needed to propose an easy way to use the system, and for the caregivers, we added more complete interfaces to allow them to manage all of the information related to their charge.

We developed a working prototype for the platform on top of the architecture described earlier; a set of two applications was developed to evaluate the feasibility of this approach, and to measure technology acceptance by the elderly.

### 2.4. Evaluation

An assessment of technology adoption and user experience of the system was performed. In the evaluation, the Technology Acceptance Model (TAM) was used [28]; this is a very useful model to predict technology usage. Its purpose is to explain the reasons for the users’ technology acceptance. This model proposes that the perceptions of usefulness and ease of use by a person in an information system are conclusive to determining their intention in using the system.

#### 2.4.1. Participants and Recruitment

The assessment of technology acceptance was performed through a pilot test with a group of 50 seniors in the State of Colima. The sample was selected by convenience sampling, per the availability of the subjects; the seniors belonged to two nursing homes at the city of Colima.

Gender distribution was equal, and 100% of participant users mentioned neither having knowledge of interactive television nor having heard about it. And also, all users said they had not used any type of technology as a reminder for their medicines or their medical appointments.

Written consent was collected from the nursing homes, according with the recommendation of the Mexican Health Department, which established that a certification issued by an official committee was required only in cases where clinical and/or biological tests would be conducted. As this study was not the case, it was sufficient to have the consent of the institutions with legal rights over the site where the study was going to be performed.

#### 2.4.2. The Intervention

First, we conducted an initial interview to learn about the context and users’ points of view regarding their habits with technology and reminders.

As a next step, the system was explained in a meeting at the nursing homes.

Later users were given a demonstration of the application using iTVCare, explaining in detail what the main goal of the application was, and its functionality.

Then, they were assigned a list of tasks to perform with the application.

Finally, participants were asked to fill out the TAM questionnaire, which is a 15-item Likert scale questionnaire to assess platform acceptance, with seven response options ranging from “1 = strongly disagree” to “7 = strongly agree”. Additionally, participants were interviewed on opinions about the platform.

## 3. Results

### 3.1. Related to Interactive TV

An interactive TV platform was developed with various features to support seniors so they could have a better quality of life.

The main characteristics of the platform are described below, as well as the activities that can be performed within them.

**Figure 2:**
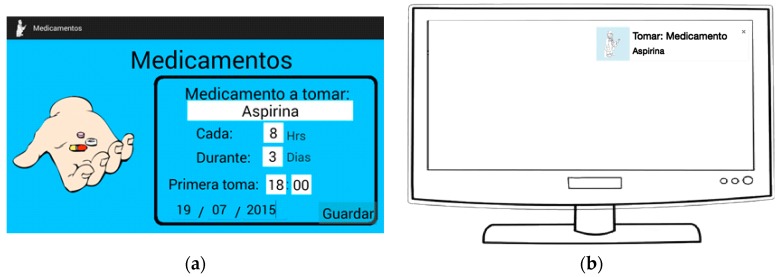
(**a**) Screen for adding medicine intake reminders; (**b**) Notification of the reminder while the older adults are watching TV.

Receive reminders for taking medication: The elderly had a user profile where they or their caregivers could set reminders for taking medication (Figure 2a), and the reminder message would appear in the television to the older adults while they were watching television (see Figure 2b). The reminder message would stay on screen until the user took action with it, the possible actions being to “close the message” or “see more details”. When choosing “see more details” a new screen appeared with the detailed information of the medication to take:

- Name of medication.
- Dose.
- Frequency of drug intake.
- Treatment duration.
- First intake.

It is important to mention, that the objective of this study was not examine medication adherence among elders. We aimed to assess the technology adoption of the proposed system to help elderly to have a better control of their daily activities. Therefore, at this stage of the project, factors such as errors in the dose of the drug or the incorrect selection of the medication were not considered.

**Figure 3:**
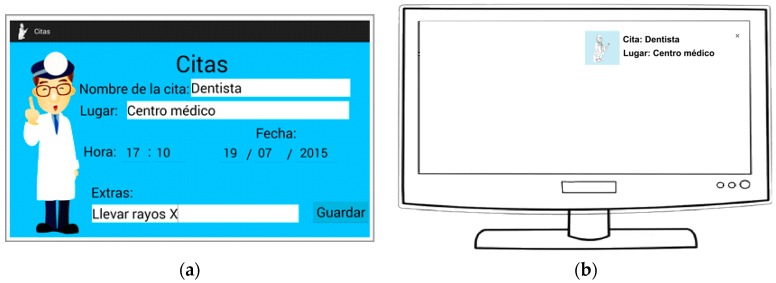
(**a**) Screen adding reminders for medical appointments; (**b**) Notification of the medical appointment while the older adults are watching TV.

Manage their agenda with their doctors: seniors or their caregivers were able to schedule medical appointments (Figure 3a); the application showed notifications for these while they were watching television, so that they remembered their medical appointments (see Figure 3b). The agenda messages stayed on screen until the user took action with it. The information included in the detailed screen for the notification are:

- Name of appointment.
- Address.
- Date.
- Time.
- Additional information.

**Figure 4:**
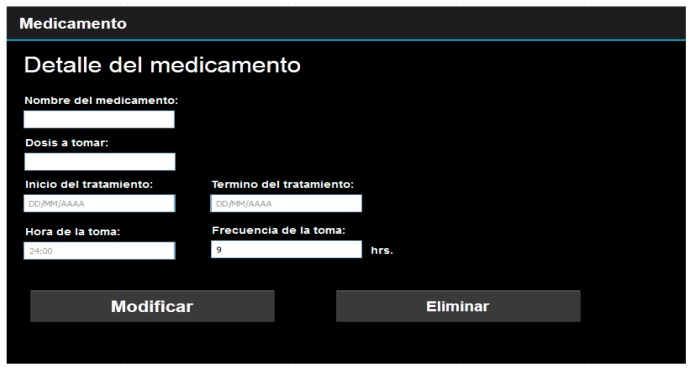
Information management by a caregiver.

Information management: A management console was available to allow the seniors’ caregivers to manage reminders and appointments entered in by the seniors (see Figure 4). This control panel allowed the caregivers more in-depth control of the information related to the data entered in iTVCare.

### 3.2. Related to the Usage of the System

From the initial interview, we characterized our users. 50% were male, 50% were female, and none of them had used technology for having medication reminders or doctor’s appointments. None of the users knew about interactive television, and 90% of users said they were willing to use technology for the reminder of their medicine and medical appointments.

The TAM questionnaire was composed of 15 items grouped into four dimensions: perceived ease of use, perceived usefulness, attitude towards the utilization of the software, and intention to use. For each item, the answer used a Likert-type scale [29]. The measurement items employed in this study were constructed from previous literature studies [28,30,31,32,33,34,35].

Table 1 presents a descriptive analysis of dimensions and their details, such as the maximum possible value and the expected notional value, which is supposed to have a high probability of acceptance. The values of the sub-scales were calculated using the sum of the maximum value possible for each item (7 on the Likert scale) in the subscale. Table A1 presents the TAM questionnaire used in our study [28,30]. All items measured on a 7-point scale ranging from 1 = “strongly disagree” to 7 = “strongly agree”.

**Figure 5:**
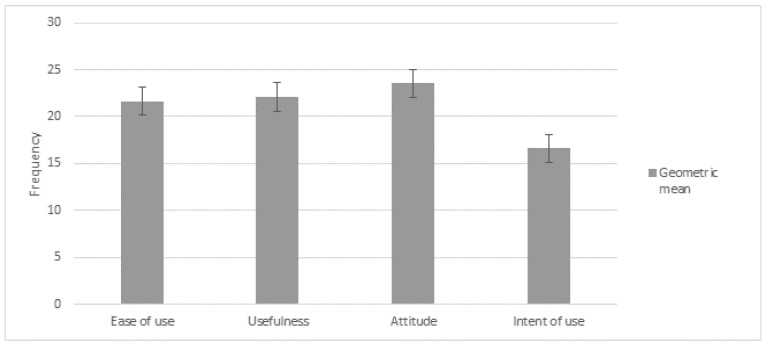
Frequency distribution of scores obtained by TAM participants.

Furthermore, an analysis of descriptive statistics is summarized in Table 2, where little variability in the responses was identified. In addition, Figure 5 shows the frequency distribution of TAM score.

As observed in the four dimensions, the mean with standard deviation did not differ from the expected values shown in Table 1. This means that the averages were not far away from the proposed expected values. This was congruent with the confidence interval of 95% given in Table 2. Therefore, in this way we could corroborate that the results were statistically similar to the expected results.

Table 3 shows the results of the statistical test, in which it was found that most users of the developed iTV platform found it easy to use and useful. Also, there was a positive attitude towards it, which could have caused greater willingness to use it.

There were significant differences between the means of the four dimensions. For this purpose, we performed a Chi-squared test, where the null hypothesis was that the averages of the four dimensions were equal, while the alternative hypothesis was that at least one of the dimensions was different. Since the *χ*^2^ calculated (0.98) was less than the value of the table ($\chi_{0.95,3}^{2}=7.8147$), for this reason we did not have reason to reject the null hypothesis, which meant that the acceptance level was statistically similar between the four dimensions.

By performing a correlation analysis, we found a correlation between the four dimensions of TAM, as shown in Table 4; as we could see, among EASE OF USE—USEFULNESS and INTENDED USE—USEFULNESS, the correlation is because most users agree that the tool is easy to use, helpful and are willing to use it. EASE OF USE—ATTITUDE, one of the factors that contributed to a correlation of 0.395, is due to the fact that 22% of users had a neutral view of the perceived ease of use, while having a good attitude. As a weakness, we identified factors that influenced the low correlation between EASE OF USE and INTENDED USE. WE found that 16% of users disagreed with the use of the tool but felt that it was easy to use; in addition, 24% had a neutral view on the use of the tool, although they considered it easy to use.

In addition, for each of the dimensions of the TAM instrument, we obtained valuable information about the opinions of our potential users. Therefore, an analysis of percentages per item in each dimension was performed, based on responses marked as “agree”, “strongly agree” and “totally agree”.

**Figure 6:**
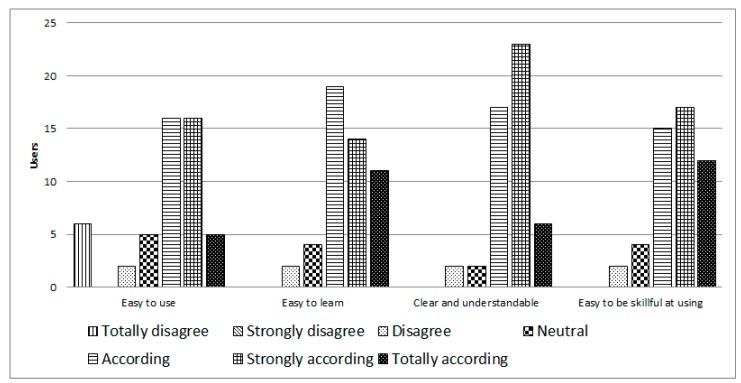
Perceived ease of use of iTVCare.

On the items of perceived ease of use (Figure 6), 74% of users believed the system was easy to use. 88% said that it was easy to learn how to operate iTVCare. 92% mentioned that their interaction with iTVCare was clear and understandable, and finally 88% said that it was easy to become skillful at using the platform.

**Figure 7:**
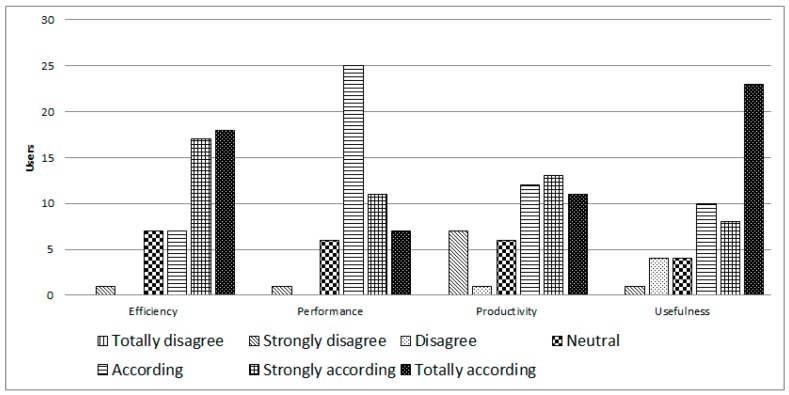
Perceived usefulness of iTVCare.

In the area of perceived usefulness (Figure 7), the results were that 84% believed it was very efficient to accomplish a task quickly, and 86% said that it would improve their performance. 72% said they would enhance their productivity, and finally 82% believed this platform could be useful.

**Figure 8:**
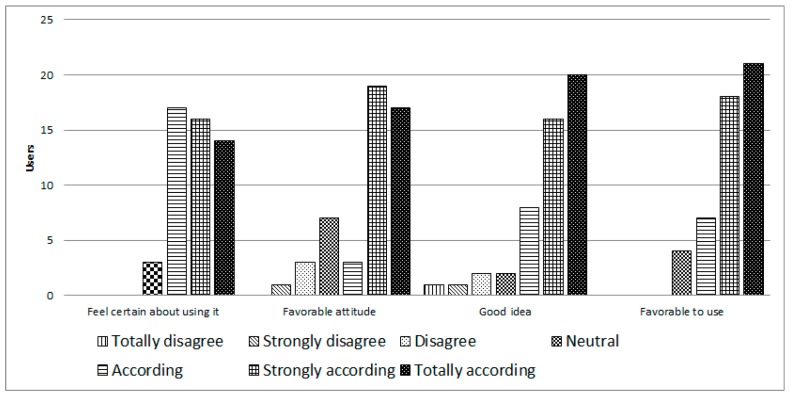
Attitudes toward use iTVCare.

Regarding the attitude towards use of iTVCare (see Figure 8), a positive response was obtained; 94% felt certain about their attitude toward using the platform, 78% had a favorable attitude toward using it, 88% believed it was a good idea to use this platform, and 92% said it was favorable to use the platform.

**Figure 9:**
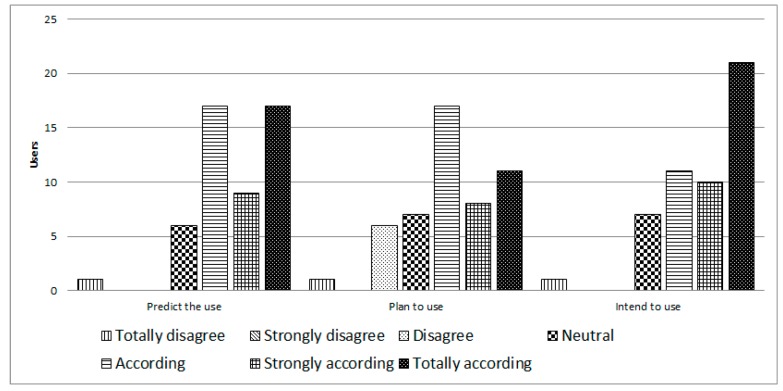
Intention of use of iTVCare.

Finally, in the category of behavioral intention (see Figure 9), 86% predicted that they would use iTVCare on a regular basis in the future if they had access to the system. 72% of users said that they planned to use the platform often, and 84% intended to use it in their daily lives (assuming they had access to the system).

### 3.3. Users Interviews

As final step, we inquired with the older adults about their general perception of iTVCare by applying a short interview with three questions:

Q1. What are the most important aspects of iTVCare?
Q2. What are the main problems of iTVCare?
Q3. Do you have other observations or suggestions for iTVCare?

The data collected from these interviews supported and complemented the findings obtained in the TAM questionnaire. From Q1, we obtained the following information: We found that 12 users made comments in which they mentioned that it was a good idea that the application was used in the television set, for instance, “using the reminders on television seem to me excellent”.

Related to the problems of Q2, 14% of the older adults said that they did not want to use the physical keyboard to type the information for the reminders, and the 10% of the participants wanted to have more images in the control panel. Finally, in the response for Q3, some of the more important comments about iTVCare were “I find the app very useful” and “the goal of the app is good”.

## 4. Discussion

This study confirmed that older adults showed a strong intention of use towards a television-based platform to provide wellbeing. Some design concepts and products are intended to improve healthy ageing for seniors, and the literature discusses different approaches, mostly based on mobile phone applications. A major contribution of this study was to validate the viability of the use of a TV set and applications (specially designed for TV) to support an ageing-friendly environment. This field is only emerging, as reported in [14], which queried Scopus and Web of Science for papers using keywords “Elderly & TV”, “Interactive & TV & Elderly”, “SmartHome”, “AAL & elderly & TV” and filtering only those related to older people and television, more than 75% of publications were released at or after 2010. Our results were in concordance with previous literature, such as the AAL FoSSIBLE project supporting social interactions through the development of smart TV applications to prevent isolation for older people living alone [36]. To meet the demand for these type of applications, the next generation of televisions must be prepared to offer interactive services and content to specific groups of users, according their characteristics and preferences [37]. However, a deeper understanding of which services may be of interest to older adults is required. Our experience in designing technology for elders indicated that this process can be very challenging; for this reason, it is very important to conduct technology acceptance studies to confirm the elderly perception of the usefulness of a technology, and to avoid the natural tendency to prefer staying with traditional ways instead of adopting technology. Unfortunately, in previous works reported in the literature, technology adoption was not the core of their objectives and, therefore, not discussed in depth. There are some studies that have evaluated the acceptance of TV-based platforms for older adults, such as Rivas Costa et al. [38], regarding the technical acceptance of a TV-based game platform to support cognitive evaluation. They also confirmed that a TV-based system has a high acceptability by older adults. 

## 5. Limitations and Future Work

The objective of this project was not to validate the clinical results of the medicine intakes of seniors. That would require a pilot test with wider parameters. In this work, we only assessed the technology adoption of a TV-based system with a set of applications to help the elderly to have a better control of their daily activities.

Although participating users were a representative sample of the State of Colima population, which at the same time is comparable to the Mexican population in middle size cities, a larger user sample would be needed to obtain significant results for an international profile.

As a general direction, we believe that designs aimed at older adults should continue to emphasize the simplification of interfaces, and make error recovery a main feature, clarifying errors with the simplest language, and explaining the process to resolve the situation.

The highly positive acceptance of iTVCare was a good starting point, but a most robust platform is required to cover the main areas where ICT are used to improve the quality of life of the elderly at home or in nursing homes. The research related to the provision of care services to elderly people can be divided into two types [39]: (a) health care, and (b) social care. We are planning to follow type (a), since we previously explored the type (b) [13]. With the current instantiation of our platform, we will develop new applications to configure a core set to demonstrate the viability of our proposal. Some planned applications are: monitoring vital signs, remote consultation, exercise at home, entertainment, serious games, and those currently in use. The application of reminders for taking medication requires updating, since during the process, some issues arose relating to medication specific particularities, for example situations where older adults did not take a medicine; when they would need different doses over the day, or reminders to take medication when they are not at home. We will review existing mechanisms and digital tools reported in the literature to support medication intakes for elderly people, and take into account important issues like intentional non-adherence, medication refill, tracking of dose skipping, or the accidental intake of another medication.

After the update, the system will be deployed at the homes of elderly users or nursing homes, and access will be granted to their caregivers and relatives.

## 6. Conclusions

We presented an electronic platform based on interactive television that helps Mexican seniors to have better control of their daily activities, such as medicine intake and medical appointments, and thus support them in achieving a better quality of life by not forgetting to take their medications and attend appointments with their doctors. This information is presented through the television that they have in their homes and use daily in an age-friendly environment. As part of the design, an evaluation of technology acceptance by the elderly was carried out and we found that most users agreed that the tool was easy to use and had good usefulness. Users had a good attitude for using it, generating a strong intention to use this platform to support their daily life activities. The aim of this study was to obtain evidence whether or not the proposed technology would be accepted by older adults, and at the same time get a response to the question if television would be a really suitable platform for technology adoption by older people and, thus, it may help to overcome the digital gap they suffer [38]. This study confirmed that older adults positively accepted iTVCare. We confirmed that the use of TV was suitable in providing simple interactions among older people and computer systems, since 92% of users mentioned that their interaction with iTVCare was clear and understandable.

## Figures and Tables

**Table 1:** Dimensions of the Technology Acceptance Model (TAM) questionnaire.

Dimension	No. of Items	Maximum Value on Likert Scale	Expected Value
Ease of use	4	28	25
Usefulness	4	28	25
Attitude	4	28	25
Intention to use	3	21	18

**Table 2:** Results of TAM application.

Dimension	Mean	Standard Deviation	Confidence Interval	
Ease of use	21.66	3.91	LI = 17.74	LS = 23.58
Usefulness	22.08	4.32	LI = 19.96	LS = 24.20
Attitude	23.56	3.46	LI = 21.86	LS = 25.26
Intention to use	16.62	3.44	LI = 14.93	LS = 18.31

LS: upper limit; LI: lower limit.

**Table 3:** Results of statistical test.

Dimension	Observed (O)	Expected (E)	(O−E)^2^	(O−E)^2^/E
Ease of use	21.66	25	11.16	0.45
Usefulness	22.08	25	8.53	0.34
Attitude	23.56	25	2.07	0.08
Intention to use	16.62	18	1.90	0.11

*χ*^2^ calculated = 0.98.

**Table 4:** Pearson correlation between the factors.

Dimension	Use	Attitude	Usefulness
Attitude	0.589	-	-
Usefulness	0.588	0.456	-
Ease of use	0.258	0.395	0.581

## Appendix A

**Table A1:** TAM questionnaire.

Subscale	Item	Measure Scale						
Perceived ease of use (Ease of use)	I would find iTVCare easy to use	1	2	3	4	5	6	7
	Learning to operate iTVCare would be easy for me	1	2	3	4	5	6	7
	My interaction with iTVCare would be clear and understandable	1	2	3	4	5	6	7
	It would be easy for me to me to become skillful at using iTVCare	1	2	3	4	5	6	7
Perceived usefulness (Usefulness)	Using iTVCare would enable me to accomplish tasks (medicine intake reminders and medical appointments) more quickly	1	2	3	4	5	6	7
	Using iTVCare would improve my performance (medicine intake reminders and medical appointments)	1	2	3	4	5	6	7
	Using iTVCare would increase my productivity (medicine intake reminders and medical appointments)	1	2	3	4	5	6	7
	I would find iTVCare useful	1	2	3	4	5	6	7
Attitude towards use (Attitude)	I feel certain about my attitude toward using iTVCare	1	2	3	4	5	6	7
	I have a favorable attitude toward iTVCare use	1	2	3	4	5	6	7
	Using iTVCare is good	1	2	3	4	5	6	7
	My using the iTVCare system is favorable	1	2	3	4	5	6	7
Behavioral intention to use (Intention to use)	I predict that I will use iTVCare on a regular basis in the future (given that I have access to the system)	1	2	3	4	5	6	7
	In the future, I plan to use iTVCare often	1	2	3	4	5	6	7
	I intent to use iTVCare (Assuming I have access to the system)	1	2	3	4	5	6	7
